# Cycling of Etk and Etp Phosphorylation States Is Involved in Formation of Group 4 Capsule by *Escherichia coli*

**DOI:** 10.1371/journal.pone.0037984

**Published:** 2012-06-04

**Authors:** Chen Nadler, Simi Koby, Adi Peleg, Austin C. Johnson, Krishna C. Suddala, Karthik Sathiyamoorthy, Bennett E. Smith, Mark A. Saper, Ilan Rosenshine

**Affiliations:** 1 Department of Microbiology and Molecular Genetics, The Hebrew University-Hadassah Medical School, Jerusalem, Israel; 2 Program in Biophysics, University of Michigan, Ann Arbor, Michigan, United States of America; 3 Department of Biological Chemistry, University of Michigan, Ann Arbor, Michigan, United States of America; University of Louisville, United States of America

## Abstract

Capsules frequently play a key role in bacterial interactions with their environment. *Escherichia coli* capsules were categorized as groups 1 through 4, each produced by a distinct mechanism. Etk and Etp are members of protein families required for the production of group 1 and group 4 capsules. These members function as a protein tyrosine kinase and protein tyrosine phosphatase, respectively. We show that Etp dephosphorylates Etk *in vivo*, and mutations rendering Etk or Etp catalytically inactive result in loss of group 4 capsule production, supporting the notion that cyclic phosphorylation and dephosphorylation of Etk is required for capsule formation. Notably, Etp also becomes tyrosine phosphorylated *in vivo* and catalyzes rapid auto-dephosphorylation. Further analysis identified Tyr121 as the phosphorylated residue of Etp. Etp containing Phe, Glu or Ala in place of Tyr121 retained phosphatase activity and catalyzed dephosphorylation of Etp and Etk. Although EtpY121E and EtpY121A still supported capsule formation, EtpY121F failed to do so. These results suggest that cycles of phosphorylation and dephosphorylation of Etp, as well as Etk, are involved in the formation of group 4 capsule, providing an additional regulatory layer to the complex control of capsule production.

## Introduction

Bacteria frequently produce polysaccharide capsules that play a significant role in various processes. Capsules contribute to biofilm formation by providing an extracellular matrix component [1], and are involved in interbacterial competition in some bacteria by forming a shield that protects from contact-dependent killing by other bacteria [2]. Capsules also play a role in virulence by several mechanisms including inhibition of phagocytosis and formation of a “decoy” for anti-bacterial peptides [3]. Furthermore, capsules were implicated in controlling the activity of surface components, such as adhesins or type III secretion apparatus, by regulated masking and unmasking of these surface structures [4], [5]. *Escherichia coli* can synthesize capsule by four distinct mechanisms categorized as groups 1 through 4. The group 1 and group 4 capsules are produced by a similar Wzy-dependent mechanism [6]. This mechanism involves the synthesis in the cytoplasm of lipid-linked repeat units consisting of several sugar residues, followed by translocation of the repeat unit to the outer leaflet of the inner membrane [7]. The translocated repeat units serve as substrates for the Wzy polymerase, which catalyzes the elongation of the polysaccharide [7]. The capsule polysaccharide (sometimes termed K-antigen) of a given *E. coli* strain is normally distinct from that of its lipopolysaccharide (LPS) O-antigen repeats. In contrast, the group 4 capsule is comprised of the same O-antigen repeats as LPS. Thus, the group 4 capsule was also termed O-antigen capsule [8].

The Wzy-mediated capsule synthesis is dependent on several additional proteins including Wzc for group 1, and Etk for group 4 capsule [8], [9], [10]. Wzc and Etk are members of the bacterial tyrosine autokinase family. Although they are transmembrane proteins, they are structurally distinct from eukaryotic transmembrane kinases. Wzc and Etk form a hairpin structure, consisting of a large periplasmic domain connected by two transmembrane helices to cytoplasmic N- and C-terminal domains [7], [11]. The cytoplasmic C-terminal domain includes a catalytic protein tyrosine kinase (PTK) domain and tyrosine-rich C-terminal “tail”, which is a substrate for the catalytic domain [6], [12]. Autophosphorylation *in trans* is required for group 1 capsule formation as mutation of the conserved Walker motif in Wzc abolished both autophosphorylation and capsule assembly [9], [10]. The Wzc and Etk genes are preceded by genes encoding low-molecular-weight protein tyrosine phosphatases (LMW-PTP), Wzb and Etp, respectively, which can use their cognate PTK as substrates. It was proposed that alternating cycles of phosphorylation and dephosphorylation of the Wzc tyrosine-rich tail are required for capsule production [7], but the specific mechanism by which this phosphorylation influences capsule production has yet to be defined. Recent crystallographic studies show that the nonphosphorylated Wzc cytoplasmic domain forms an octamer that is disrupted following phosphorylation, potentially causing the Wzc periplasmic domains to cycle between two states [13]. Furthermore, Wzc and related proteins also promote polysaccharide synthesis by phosphorylation of substrates other than the tyrosine-rich tail in order to activate sugar nucleotide precursor biosynthesis or activate glycosyl transferases [14].

LMW-PTPs similar to Wzb and Etp frequently occur in prokaryotes and eukaryotes. These enzymes characteristically function via a conserved cysteine, which serves as nucleophile, and an invariant aspartic acid located in a DPY or DPYY conserved loop. The eukaryotic LMW-PTPs are involved mainly in signal transduction and their activity is subject to stringent regulation. For instance, oxidation of the catalytic cysteine thiol inhibits their enzymatic activity in a reversible manner [15]. The activity of eukaryotic LMW-PTPs are also regulated by phosphorylation of tyrosine residues in the DPYY loop (Tyr131 and Tyr132). Tyr131 phosphorylation may modulate its enzymatic activity [16], [17], and Tyr132 phosphorylation may provide substrate specificity [18]. The tyrosine residue in the DPY loop of Wzb/Etp was also implicated in substrate recognition [19].

In this report, we analyze the involvement of Etk and Etp in synthesis of group 4 capsule by enteropathogenic *E. coli* (EPEC). We show that cycling of Etk phosphorylation and dephosphorylation by Etp is required for formation of group 4 capsule. We further show that Etp is also subject to tyrosine phosphorylation, that Etp's phosphorylated residue is Tyr121, and, like Etk, Etp also cycles between phosphorylated and unphosphorylated forms. Mutagenesis data, both *in vivo* and *in vitro*, support the notion that tyrosine phosphorylation of Etp is required for the formation of group 4 capsule. These findings elucidate an additional regulatory layer of capsule synthesis.

## Results

### Etk kinase activity is required for formation of group 4 capsule

**Figure 1:**
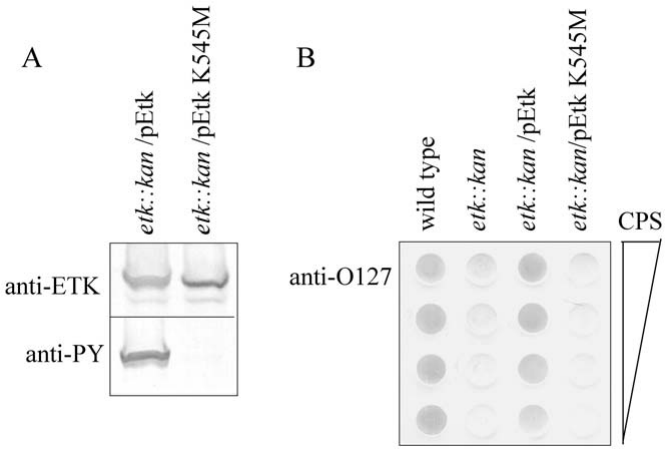
Etk K545M mutant failed to support capsule production. EPEC strains, including wild type, *etk::kan* mutant and the mutant complemented with plasmids pOI277 (pEtk) or pMS3239 (pEtk K545M), were grown under conditions that allowed capsule production. After harvesting, proteins and capsular polysaccharide were extracted. (A) The amount of Etk and phosphorylated Etk were assessed by Western blot using anti-Etk and anti-phosphotyrosine (anti-PY) antibodies. Each lane is labeled with the strain (above blot) and antibody (on left). (B) Purified capsular polysaccharide extracted from bacteria was serially diluted to generate a dot blot that was developed with anti-O127 antibody. Wild-type EPEC and *etk* mutant were used as positive and negative controls, respectively. The strains are indicated above each serial dilution.

We first tested whether the Etk catalytic activity is required for production of group 4 capsule. To this end, we constructed a plasmid expressing inactive Etk. The invariant Lys 545 of Etk, which is essential for kinase activity, was replaced by methionine. Plasmids expressing wild-type Etk or the mutated Etk (EtkK545M) were introduced into EPEC, in which the native *etk* gene was inactivated (*etk::kan*), and the ability of the plasmids to restore capsule production was tested. First, we confirmed that the bacteria equally expressed both Etk and EtkK545M and that EtkK545M had lost its autokinase activity (Fig. 1A). Next, we extracted polysaccharides from the bacteria, purified the capsular polysaccharide without any LPS contamination, and probed by dot blot with anti-O127 antibody. The results confirmed that wild type EPEC produced capsular O127 polysaccharide, but the *etk::kan* mutant failed to do so. Capsule production was restored by a plasmid expressing Etk, but not by the plasmid that expressed EtkK545M (Fig. 1B). These results suggested that the catalytic activity of Etk is essential for production of group 4 capsule.

### Etp dephosphorylates Etk *in vivo* and is tyrosine phosphorylated

**Figure 2:**
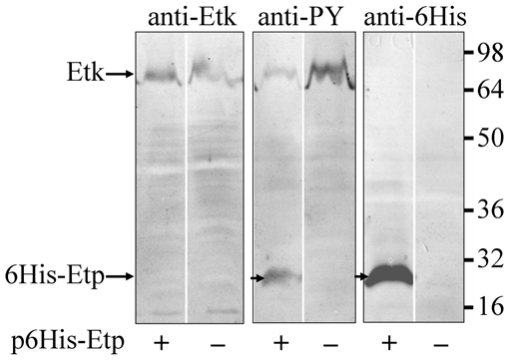
Etp promotes Etk dephosphorylation and is tyrosine phosphorylated *in vivo*. EPEC strains transformed, or not transformed, with plasmid pAP406 (p6His-Etp) expressing 6His-Etp, were grown overnight in LB at 20°C without shaking. The bacteria were harvested, and the extracted proteins were separated for Western blot analysis with anti-Etk, anti-phosphotyrosine (anti-PY), and anti-6His antibodies. The corresponding antibody is indicated above each panel. Also indicated are the location of Etk and 6His-Etp and the molecular size markers (at the right). Whether a given strain contained the pEtp plasmid is indicated below each lane.

To test if Etp can dephosphorylate Etk *in vivo*, we transformed EPEC with a plasmid encoding 6His-Etp (pAP406) and expressed the recombinant Etp in LB at 20°C without shaking (conditions that enhanced Etp solubility). As control, we used wild type EPEC that did not express the recombinant Etp. Proteins were extracted from the two strains and analyzed by Western blot with anti-Etk, anti-6His and anti-phosphotyrosine (anti-PY) antibodies. The results show a clear decrease in tyrosine phosphorylated Etk upon Etp overexpression (Fig. 2), suggesting that Etp dephosphorylates Etk *in vivo*. Intriguingly, we noticed that the anti-PY antibody also reacted with the recombinant Etp (Fig. 2), indicating that some of the Etp was tyrosine phosphorylated. We also noted that the blotted Etp regained some of its acid-phosphatase activity during the blot development to catalyze autodephosphorylation (Fig. S1). To reduce the autodephosphorylation activity, blots were developed at pH 8 to inhibit Etp (Fig. S2). To exclude the possibility that the anti-PY antibody recognized Etp by nonspecific cross-reaction, we included the PY competitive inhibitor phenylphosphate that abolished the antibody interaction with Etk (Fig. S3). Taken together, our results show that Etp becomes tyrosine phosphorylated *in vivo* and that it catalyzes autodephosphorylation.

### The Etp phosphorylated residue is Tyr121

**Figure 3:**
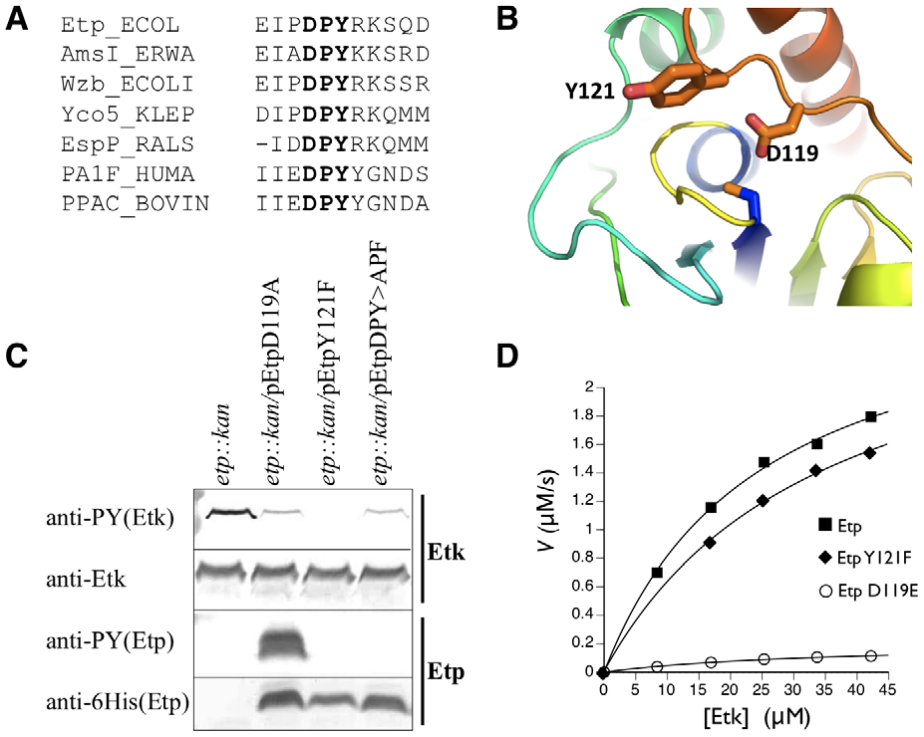
Identification of the phosphorylated Etp residue. (A) The region containing the DPY motif (in bold) of Etp is compared with the corresponding region of several members of the LMW-PTP family. They include AmsI of *Erwinia amylovora*, the *E. coli*-encoded Etp paralog Wzb, Yco5 of *Klebsiella pneumoniae*, EspP of *Ralstonia solanacearum*, the human PA1F protein, and the bovine PPAC protein encoded by the ACP1 gene. Numbering is according to the Etp sequence. (B) Homology model of the Etp structure in the absence of bound substrate based on the NMR structure of Wzb [19]. Side chains Tyr121 and Asp119 are shown in sticks. The phosphate binding loop (yellow) and catalytic cysteine C13 are in the center. (C) EPEC mutant Δ*etp::kan* was transformed with plasmids expressing different Etp mutants all of which had C-terminal 6His tags. Proteins were extracted from the different cultures and were subjected to Western blot analysis with anti-Etk, anti-6His and anti-PY antibodies as indicated. The corresponding strain is indicated above each of the lanes. (D) *In vitro* kinetics of Etp and variants with phosphorylated MBP-Etk as substrate. The graph plots the rate of inorganic phosphate produced versus substrate concentration for Etp, EtpY121F, and EtpD119E. Kinetic constants are in Table 1.

Comparison of Etp to other LMW-PTPs associated with polysaccharide production highlighted only one conserved tyrosine residue, Tyr121, located in the conserved DPY motif (Fig. 3A, B) that also included the essential catalytic acid Asp119. To test whether Tyr121 is the Etp phosphorylation site, we constructed plasmids expressing Etp mutants. Prior to plasmid construction, we determined the actual N-terminus of Etp, since early versions of the EPEC genome sequence annotated the *etp* ORF starting at a GTG codon, 5 codons prior to an ATG codon. To resolve which codon was the native translation start site, we cloned *etp* including the 80 bp upstream of the putative GTG initiation codon that contained the *etp* native ribosomal binding site. In addition, we added a hexahistidine (6His) tag to the 3′ end of the *etp* ORF followed by a stop codon. The generated plasmid pCNY506, encoding Etp-6His, was introduced into *E. coli* and the expressed Etp-6His was purified by affinity chromatography. The native N-terminal sequence of the purified protein was determined to be MAQLKFNSILVVZ. These results indicated that Etp translation starts at the first ATG of the ORF rather than the GTG. We thus termed the ATG encoded residue as Met1. We further used this vector to generate Etp mutants in the DPY motif.

We next expressed the constructed Etp mutants, EtpD119A and EtpY121F, in the EPEC Δ*etp::kan* strain, and examined whether they were tyrosine phosphorylated. Our results showed that EtpD119A was deficient in phosphatase activity, and became hyperphosphorylated due to Etp's inability to dephosphorylate itself (Fig. 3C). In contrast, EtpY121F was completely unphosphorylated suggesting that Y121 is the phosphorylated residue. EtpY121F maintained its phosphatase activity as indicated by its capacity to dephosphorylate Etk *in vivo* (Fig. 3C). To confirm this catalytic activity, we measured the *in vitro* ability of Etp and EtpY121F to dephosphorylate the cytoplasmic catalytic domain of phosphorylated Etk. Again, Etp and EtpY121F exhibited similar activity (Fig. 3D and Table 1). To further exclude the possibility that EtpY121F is not phosphorylated simply due to increased autodephosphorylation *in vivo*, we constructed a plasmid expressing Etp where both D119 and Y121 were mutated (to give EtpDPY>APF). This protein was not phosphorylated although it was deficient in phosphatase activity as indicated by reduced dephosphorylation of Etk (Fig. 3C). Taken together, these results indicated that Etp Tyr121 becomes phosphorylated *in vivo*.

**Table 1:** *In vitro* kinetics of MBP-Etk dephosphorylation by 6His-Etp and 6His-EtpY121F.

	*k*_cat_ sec^−1^	*K*_M_ (µM)	*k*_cat_/K_M_ (sec µM)^−1^ (×10^−3^)
Etp wild type	2.84	24.8	115
EtpY121F	2.86	34.9	82

**Figure 4:**
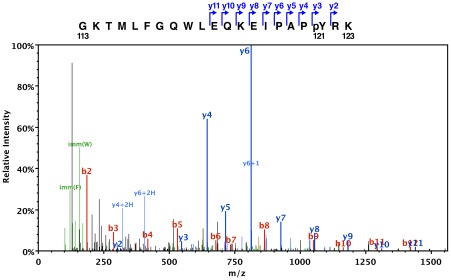
ESI tandem mass spectrometry confirms the presence and location of the phosphorylated tyrosine. The ESI-MS/MS fragmentation spectrum of Etp peptide GKTMLFGQWLEQKEIPAP(pY)RK (residues 113–123, pY is the phosphorylated tyrosine). The parent peptide was a +4 H ion with mass 2599.29 amu (−0.0134 amu from predicted size). The schematic at the top of the figure shows the identified **y** fragments referred to in the spectra. All of the **y** fragments include the peptide's C-terminus and have masses consistent with the phosphorylated Y121 (pY) residue. For example, the m/z for fragments **y3** and **y2** are 546.2 and 303.2, respectively. The difference 243 is the exact mass of a phosphotyrosyl residue. The labeled **b** fragments originate at the N-terminus of the peptide but do not include the pY residue.

We noticed that Etp phosphorylation was not restricted to EPEC and was also observed in other *E. coli* strains. We took advantage of this observation to further examine the finding that Tyr121 was phosphorylated. EtpD119A was expressed from pAJ0046 in *E. coli* BL21(DE3) and purified. Western blotting with anti-phosphotyrosine antibody 4G10 (Millipore/Upstate) confirmed that the protein was hyper-phosphorylated when compared to wild type Etp (data not shown). The purified protein was analyzed by electrospray tandem mass spectrometry (LC-MS/MS) by MS Bioworks, LLC (Ann Arbor MI). The size of peptides that included Tyr121 as well as their fragmentation patterns conclusively showed the presence of a phosphorylated tyrosine (+80 a.m.u.) corresponding to position 121 of the native protein (Fig. 4). This was observed in 31 of 64 spectra of peptides containing Tyr121. Fig. 4 is a typical spectrum showing C-terminal fragments before (y3) and after (y2) the phosphorylated Tyr121. No tyrosine phosphorylation was observed elsewhere in the protein.

### EtpY121F is deficient in supporting capsule formation

**Figure 5:**
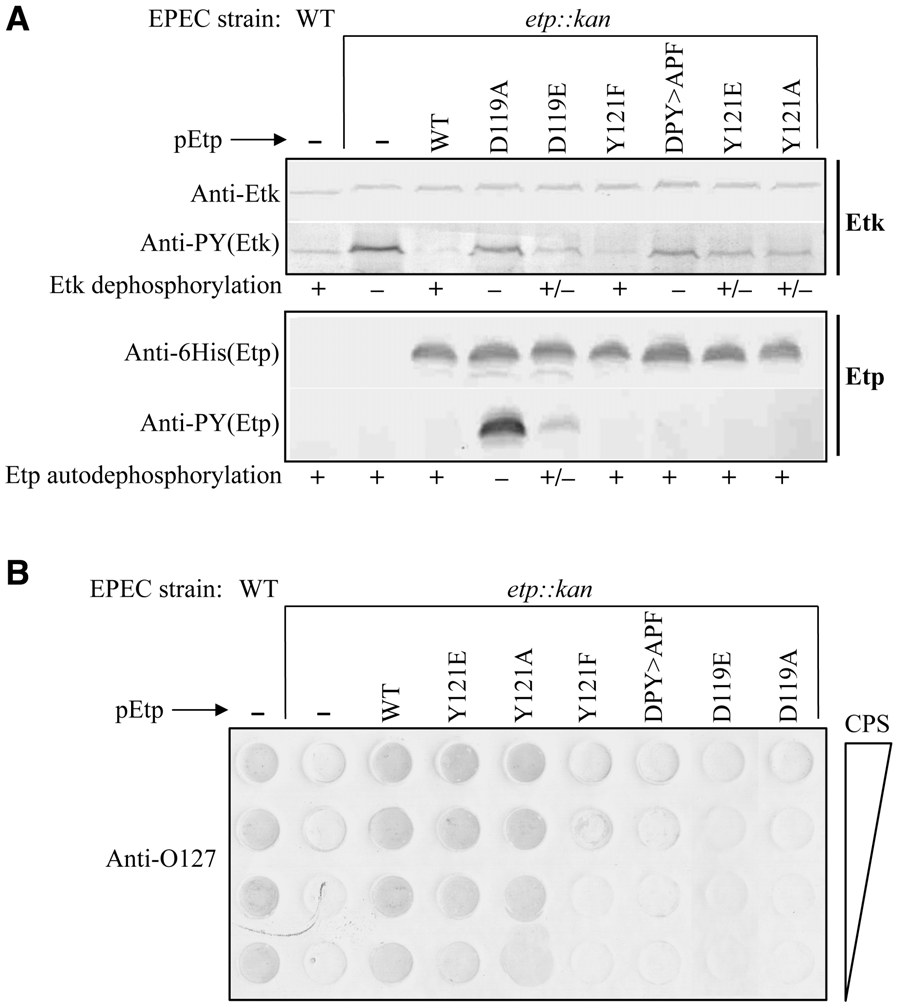
EtpY121F retained phosphatase activity but failed to support capsule formation. (A) Proteins were extracted from wild-type EPEC, the Δ*etp::kan* mutant, or mutant complemented with plasmids derived from pEtp (pCNY506) and expressing different Etp variants with C-terminal 6His tags. The levels of Etk, phosphorylated Etk, recombinant Etp, and phosphorylated Etp were determined by Western blot analysis using anti-Etk, anti-6His, and anti-PY antibodies. The strain and the complementing mutant of Etp are indicated above the blots and the antibodies used in the immunoblots are indicated on the left side. The levels of Etk dephosphorylation and Etp autodephosphorylation are indicated below the blots. (B) Capsule polysaccharide was extracted and purified from the same strains presented in (A). Two-fold serial dilutions of purified capsule were dotted on PVDF and developed with anti-O127 antibody. The identity of the strain and complementing plasmid are indicated above the blot and direction of capsule polysaccharide dilution is indicated at the right side of the blot.

We next tested whether Etp phosphorylation on Tyr121 plays a role in capsule formation. The general strategy was to generate different point mutations in a plasmid encoding Etp with 6His at its C-terminus. The different constructed plasmids were then tested for the ability to complement EPEC Δ*etp::kan* by restoring Etk dephosphorylation, autodephosphorylation and rescue of capsule formation. The Etp mutants included i) EtpY121F, mimicking the Etp unphosphorylated form; ii) EtpY121E, mimicking the Etp phosphorylated form; and iii) EtpY121A, lacking both the charge and phenolic ring. As negative controls we included in this analysis EtpD119A and EtpD119E, which exhibit severe and moderate attenuation in phosphatase activity, respectively. As an additional control we used EtpDPY>APF. Similar expression levels of all Etp mutants were confirmed by Western blots with anti-6His antibody (Fig. 5A).

Our results showed that the phosphatase activity of EtpD119A and EtpDPY>APF were strongly attenuated as judged by their inability to dephosphorylate Etk or catalyze autodephosphorylation (EtpD119A) (Fig. 5A). EtpD119E activity was partially attenuated as seen by partial autodephosphorylation and decreased dephosphorylation of Etk. Unlike EtpD119A that was inactive in the *in vitro* Etp dephosphorylation assay (data not shown), EtpD119E had low activity (Fig. 3D), consistent with the *in vivo* results. These three Etp mutants also failed to produce significant capsule polysaccharide (Fig. 5B). Surprisingly, the mutant expressing EtpY121F, which *in vivo* and *in vitro* showed similar phosphatase activity towards Etk as wild-type Etp (Fig. 5A, 3D and Table 1), also failed to produce capsule (Fig. 5B). Etp containing other substitutions of Tyr121, including EtpY121E and EtpY121A, exhibited both phosphatase activity and rescue of capsule production, albeit at efficiencies slightly lower than that of wild type Etp (Fig. 5).

Taken together, our results indicate that the catalytic activity of Etp is essential for production of O127 group 4 capsule, probably due to the need to dephosphorylate Etk. Furthermore, the results show that Tyr121 *per se*, is not required for capsule formation or for phosphatase activity. However, expression of EtpY121F, which mimics the nonphosphorylated form of Etp, inhibits capsule formation by a mechanism unrelated to the phosphorylation state of Etk. Assuming that EtpY121F and EtpY121E mimic the dephosphorylated and phosphorylated forms of Etp, respectively, our results suggest that nonphosphorylated Etp inhibits capsule formation and Etp phosphorylation on Tyr121 alleviates this inhibition.

### Etk and Wzc are not essential for Etp phosphorylation

**Figure 6:**
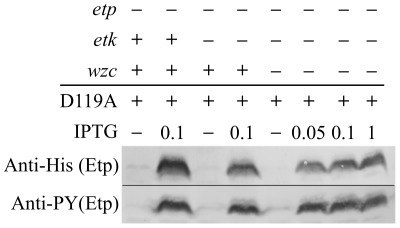
Etk and Wzc are not essential for Etp phosphorylation. EtpD119A was expressed in different EPEC strains with deletions in the *etp* gene and the kinase genes *etk* and *wzc*. Proteins were extracted and the amount and phosphorylation state of Etp were tested by Western blot using anti-6His and anti-PY antibodies, respectively. The presence of intact genes is indicated above the lanes.

We next tested whether Etk is the tyrosine kinase that phosphorylates Etp *in vivo*. We transformed EPEC containing an inactivated *etk* gene (EPEC *etk::kan*) and the EPEC double mutant *etk::kan, etp::cm* with a plasmid expressing EtpD119A, and compared EtpD119A phosphorylation between the two strains. The results showed that EtpD119A becomes phosphorylated even in the EPEC *etk::kan* mutant (Fig. 6). The only other known PTK encoded by *E. coli* strains including EPEC is Wzc. We thus constructed the triple mutant *etp::cm, etk::kan, wzc::tet* and tested the capacity of this strain to phosphorylate EtpD119A. Notably, also in this case the EtpD119A remained phosphorylated (Fig. 6). Taken together our results indicate that neither Etk nor Wzc is essential for Etp phosphorylation and that the identity of the kinase that phosphorylates Etp remains unknown.

## Discussion

The *etp* and *etk* genes are the last genes of a group 4 capsule operon found in most *E. coli* strains that includes seven genes: *gfcABCDE*, *etp* and *etk* (formerly *ymcDCBA, yccZ, yccY* and *yccC*, respectively) [8]. The Etk autokinase, in analogy to its close paralog Wzc, is predicted to function as a component of a larger protein complex that transverses the bacterial envelope and forms a polysaccharide secretion system [7]. Wzc, and probably also Etk, form a tetrameric [20] or octameric [13] complex that crosses the inner membrane and interacts through its periplasmic domains with the outer membrane protein octamer Wza, a member of the OPX (outer membrane polysaccharide export) family [7]. Wza oligomers are both in contact with Wzc and form a channel-like structure in the outer membrane [7]. Since Etk and GfcE are very similar to Wzc and Wza (51% and 64% sequence identity, respectively), it is likely that Etk and GfcE form a complex similar to that formed by Wzc and Wza. Additional components in these systems are Wzb (for group 1) or Etp (for group 4 capsule), both LMW-PTPs that target Wzc and Etk, respectively [9], [10, this study]. Our results and results of others suggest that phosphorylation and dephosphorylation cycling of at least the kinase carboxyl terminus is crucial for the proper functioning of the Wzc-dependent system and thus for formation of group 1 capsule [7]. In this report we show for the first time that Etp dephosphorylates Etk *in vivo* and that mutations which abolish either the catalytic activity of Etk or Etp result in a deficiency in the synthesis of group 4 capsule. These results also suggest that alternating cycles of Etk phosphorylation and dephosphorylation are required for group 4 capsule formation. These new data extend the similarity between the mechanisms involving the synthesis of group 1 and group 4 capsules.

Unexpectedly, we noticed that Etp became tyrosine phosphorylated *in vivo*. Further analysis defined Tyr121, located within a DPY motif, as the phosphorylated residue. Interestingly, Tyr121 was implicated in substrate recognition by Wzb, the Etp close homolog [19]. Furthermore, in eukaryotic LMW-PTPs, Tyr131 and Tyr132 within the DPYY motif, which corresponds to the DPY motif of Etp, are subjected to phosphorylation [16]. These eukaryotic LMW-PTPs, and likely also Etp, may catalyze autodephosphorylation by forming transient dimeric structures [21]. Phosphorylation of eukaryotic LMW-PTP was previously implicated in regulation of its activity [16], [17].

Biochemical analysis of the intrinsically unstable tyrosine phosphorylation of Etp is challenging. We thus adopted an alternative approach to deepen our insight as to the role of Etp phosphorylation by generating mutants that might mimic either the Etp phosphorylated form (EtpY121E) or unphosphorylated form (EtpY121F), or test the importance of the phenolic ring of Tyr121 (EtpY121A). Notably, all Etp Y121 mutants exhibited PTP activity *in vivo* similar to that of the wild type Etp, but while EtpY121E and EtpY121A also supported capsule production, EtpY121F failed to do so. These results indicate that like Etk, Etp cycles between phosphorylated and dephosphorylated forms during capsule production. Furthermore, the results suggest that the unphosphorylated Etp may inhibit capsule formation, regardless of its phosphatase activity, and that this inhibition is alleviated upon Etp phosphorylation.

**Figure 7:**
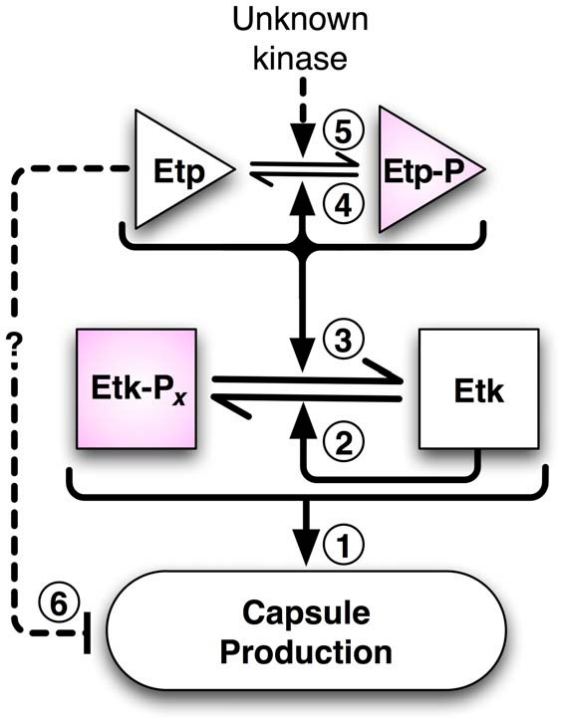
A model for the role of Etp and Etk in capsule formation. Cycling of Etk phosphorylation and dephosphorylation are required for capsule production (arrow **1)**. This cycling is mediated by the autokinase and autophosphatase activities of Etk and Etp, respectively **(**arrows **2** and **3**). Etp also cycles between phosphorylated and unphosphorylated forms and this cycling is catalyzed by Etp autodephosphorylation (arrow **4**) and a yet to be defined kinase (arrow **5**). Etp cycling *per se* is not required for capsule formation. However, based on our results we hypothesize that the unphosphorylated Etp has an inhibitory effect on capsule formation (arrow **6**), which can be removed by Etp phosphorylation.

Based on our results we propose the model shown in Fig. 7. This model and our study raise several new questions. First, why does EtpY121F with near wild-type catalytic activity inhibit capsule formation? One possibility is that the Y121F substitution alters the enzyme's substrate specificity. If this is the case, the affected substrate is likely not Etk, since our results showed that EtpY121F dephosphorylated Etk as efficiently as wild-type Etp. UDP-glucose dehydrogenase (Ugd) has been described to be a substrate of Wzc/Wzb proteins in *E. coli* [14]. By analogy, if Etp dephosphorylates Ugd, the Y121F substitution could affect substrate recognition of Ugd. Why Etp phosphorylation alleviates this inhibitory effect must also be addressed. A second possibility is that Phe121 affects the interaction of EtpY121F with other Etp molecules or with another protein affecting its function. Interestingly, a *Bacillus subtilis* LMW-PTP, whose sequence contains Phe in place of Etp's Tyr121, was observed by NMR to oligomerize at sub-millimolar concentrations [21].

How Etp is phosphorylated is another important unanswered question. Etk and Wzc are not essential for Etp phosphorylation, but might contribute to this activity. Nevertheless, our data suggest that another putative *E. coli* kinase phosphorylates Etp, adding an additional layer of regulation to capsule production. A likely candidate might be a bacterial P-loop ATPase, like MinD, ParA, or ArsA, that share catalytic and structural similarities to Etk and Wzc [12].

## Materials and Methods

### Bacterial strains and plasmids

Table 2 shows the EPEC strains used in this study. Bacteria were grown in Luria Broth (LB), or in LB without NaCl where indicated. Isopropyl β-d-1-thiogalactopyranoside (IPTG) (concentration according to text), ampicillin (Amp) 100 µg/ml, kanamycin (Kan) 40 µg/ml, and chloramphenicol (Cm) 25 µg/ml were added to the growth media as needed. Bacterial strains were constructed using the lambda Red method as described [22]. The plasmids and primers used in this study are listed in Table 3 and Table 4, respectively. To generate pKS2084, the *etk* reading frame encoding the catalytic domain (residues 446–726) was amplified by PCR and cloned into pSJ8 previously digested with BamHI and XhoI. The resulting plasmid encoded the maltose-binding protein (MBP) followed by an 8×His tag, TeV protease site, and Etk residues 446–726. Etp and Etk point mutations were generated with the QuikChange Site-Directed Mutagenesis Kit (Stratagene). In all cases DNA sequencing verified the presence of the desired mutations and lack of other mutations.

**Table 2:** Enteropathogenic *E. coli* strains.

EPEC strains	Description	Reference
E2348/69	EPEC O127:H6, a clinical isolate	[23]
AP2417	E2348/69 Δ*etp::kan*	[8]
AP1104	E2348/69 Δ*etp::cm*	[8]
OI899	E2348/69 *etk::kan*	[11]
AP1417	E2348/69 Δ*etp::cm etk::kan*	This study
CNY3101	E2348/69 Δ*etp::cm etk::kan* Δ*wzc::tet*	This study

**Table 3:** Plasmids.

Name (stock#)	Description	Reference/source
pOI277	pACYC184 containing *lacI* ^q^ and 6*his-etk* under control of the *tac* promoter	[11]
pMS3239	pOI277 expressing the K545M inactive mutant	This study
pSJ8	pET21a with MBP-8His-(TeV protease cleavage site)- upstream of cloning site	Zhaohui Xu
pKS2084	pSJ8 expressing MBP-8His-TeV-Etk(446–726), amplified from pOI277 with primers 207 and 45	This study
pET28b	Expression vector with T7 promoter	Novagen
pMS0125	pET28b expressing 6His-TeV-Etp	This study
pAJ0046	pMS0125, expressing 6His-EtpD119A	This study
pAJ0049	pMS0125, expressing 6His-EtpY121F	This study
pAJ2090	pMS0125, expressing 6His-EtpD119E	This study
pSA10	Expression vector with *tac* promoter	[24]
pCNY506 (2723)	pSA10 carrying *etp*, with 80 bp upstream region and 6His at C-terminus.	This study
pCNY507 (2790)	pCNY506, expressing EtpD119A	This study
pAJ3242	pCNY506, expressing EtpD119E	This study
pCNY508 (2792)	pCNY506, expressing EtpY121F	This study
pCNY512 (3139)	pCNY506, expressing EtpY121E	This study
pCNY513 (3152)	pCNY506, expressing EtpY121A	This study
pCNY510 (2967)	pCNY506, expressing EtpD119A, Y121F	This study
pAP406	pQE31 containing *lacI* ^q^ and expressing 6His*-*Etp	[8]
pKD4	Template for amplification of Kan cassette	[22]
pKD46	Temperature-sensitive plasmid encoding lambda Red genes	[22]

**Table 4:** Oligonucleotide primers used in this study.

No.	Primer Sequence (5′ to 3′)*	Use
424	GCGAATTCCCGCTGGCTCGTTGGAACCG	Forward primer for creating pCNY506
425	GCGTCGACTTAGTGATGGTGATGGTGATGCCGGCTGAGGCGCTTCGC	Reverse primer for creating pCNY506
225	CTATCCGCATATGGCCCAACTAAAATTTAAC	Forward primer for creating pMS0125
226	CGACTCGAGTTACCGGCTGAGGCGCTTCG	Reverse primer for creating pMS0125
207	CGTGGGATCCCGTGCGATGTTGCGTCGTG	Forward primer for creating pKS2084
45	GATCTCGAGTCACTCTTTCTCGGAGTAACTATAAC	Reverse primer for creating pKS2084
263	ACCGTCAATGGTATGGTC	Forward primer for creating *etk::kan* in AP1417
264	CGTTTTACCACTGTCTGG	Reverse primer for creating *etk::kan* in AP1417
475	CGGGAAACGTTTGCAGCGGTGTACACATTACTTGAACGGTCTGCCCGCCAcaagagggtcattatatttcg	Forward primer for creating Δ*wzc*::*tet*
476	AGGTAGGTCGGATAAGATGCGTCAGCATCGCATCCGACAGCGGATGTCGactcgacatcttggttaccg	Reverse primer for creating Δ*wzc*::*tet*
480	GAAAGAGATCCCGGCTCCCTATCGTAAAAG	Forward primer for EtpD119A substitution in pCNY507
481	CTTTTACGATAGGGAGCCGGGATCTCTTTC	Reverse primer for EtpD119A substitution in pCNY507
298	GATCCCGGATCCCTTTCGTAAAAGTCAGG	Forward primer for EtpY121F substitution in pCNY508
299	CCTGACTTTTACGAAAGGGATCCGGGATC	Reverse primer for EtpY121F substitution in pCNY508
460	GAAAGAGATCCCGGCTCCCTTTCG TAAAAG	Forward primer for EtpD119A, Y121F substitutions in pCNY510
461	CTTTTACGAAAGGGAGCCGGGATC TCTTTC	Reverse primer for EtpD119A, Y121F substitutions in pCNY510
437	GAGATCCCGGATCCCGAGCGTAAAAGTCAGGACG	Forward primer for EtpY121E substitution in pCNY512
438	CGTCCTGACTTTTACGCTCGGGATCCGGGATCTC	Reverse primer for EtpY121E substitution in pCNY512
493	GAGATCCCGGATCCCGCGCGTAAAAGTCAGGACG	Forward primer for EtpY121A substitution in pCNY513
494	CGTCCTGACTTTTACGCGCGGGATCCGGGATCTC	Reverse primer for EtpY121A substitution in pCNY513
58	CAGAAAGAGATCCCGGCTCCCTATCGTAAAAGTCAGG	Forward primer for EtpD119A substitution in pAJ0046
59	CCTGACTTTTACGATAGGGAGCCGGGATCTCTTTCTG	Reverse primer for EtpD119A substitution in pAJ0046
54	GAGATCCCGGATCCCTTTCGTAAAAGTCAGGACG	Forward primer for EtpY121F substitution in pAJ0049
55	CGTCCTGACTTTTACGAAAGGGATCCGGGATCTC	Reverse primer for EtpY121F substitution in pAJ0049
49	CAGAAAGAGATCCCGGAGCCCTATCGTAAAAGTCAGG	Forward primer for EtpD119E substitution in pAJ2090, pAJ3242
48	CCTGACTTTTACGATAGGGCTCCGGGATCTCTTTCTG	Reverse primer for EtpD119E substitution in pAJ2090, pAJ3242

* Underlined sequences represent restriction enzyme sites, and lower case sequences are regions complementary to the template plasmid containing the antibiotic cassette.

### Purification of Etp-6His and N-terminal sequencing

An overnight culture of *E. coli* XL1B containing pCNY506, expressing Etp-6His from the *etp* native promoter, was diluted 1∶100 in 500 ml LB and incubated at 37°C with shaking. Upon reaching an optical density (OD_600_) of 0.6, the culture was centrifuged (4,100 g, 7 min) and the pellet was re-suspended in 4 ml lysis buffer containing 6 M guanidinium hydrochloride, 20 mM Tris-HCl pH 8, 100 mM NaCl, 0.5% Triton-X100 and 100 ug/ul PMSF. After 30 min of incubation the lysate was sonicated and clarified by repeated centrifugations (36,400 g, 7 min). The clear lysate was subjected to metal affinity chromatography using 200 µl of Talon beads (Clontech Inc.). Etp-containing fractions were electrophoresed on a 15% SDS-PAGE gel, transferred to PVDF membrane (Whatman, Inc.), and stained with Coomassie Blue. The gel band containing Etp-6His was excised and sequenced by Edman degradation with a Perkin Elmer (Applied Biosystems) model 492 sequencer system.

### Immunoblot analysis

Samples were separated by SDS-PAGE and transferred to nitrocellulose membrane. The membrane was washed briefly with TBS-pH 8 (150 mM NaCl, 20 mM Tris-HCl pH 8) and blocked for two hours with 5% bovine serum albumin (BSA, Sigma) and 0.01% Tween-20 (Sigma) in TBS-pH 8. When needed, blocking solutions of different pH were used as indicated. The blots were then developed with monoclonal anti-PY (PT66 Sigma), anti-6His (clone 6H, Sigma), anti-OK127 (Statens Serum Institut, Denmark) as anti-O127, or polyclonal rabbit anti-Etk antibodies [11] diluted in TBS-pH 8 containing 1% BSA. Anti-mouse IgG or anti-rabbit IgG conjugated with alkaline phosphatase (Sigma) or horseradish peroxidase (Promega) were used as secondary antibodies. Detection was done with NBT/BCIP (Promega) or ECL substrate (Promega), respectively.

### Mass spectroscopy analysis

To prepare His6–Etp(D119A) for mass spectroscopy, *E. coli* BL21(DE3) transformed with plasmid pAJ0046 was grown in Terrific Broth at 37°C, cooled for 30 min at 20°C, then induced with 0.2 mM IPTG. Cells were lysed by sonication in 300 mM NaCl, 2 mM TCEP, 2 mM sodium orthovanadate, 50 mM sodium phosphate, pH 7.4. Following metal affinity chromatography of the soluble fraction with TALON Superflow resin (Clontech), the purified protein was run on a 15% SDS-PAGE gel and stained with Thermo GelCode™ Blue Safe stain. The major band corresponding to His6-Etp(D119A) was excised, washed with 25 mM ammonium bicarbonate followed by acetonitrile, reduced with 10 mM dithiothreitol at 60°C, and alkylated with 50 mM iodoacetamide at 22°C. The sample was then digested with sequencing grade trypsin (Promega) at 37°C for 4 hr, quenched with formic acid, and the supernatant was analyzed directly without further processing. The sample was analyzed by nano LC/MS/MS at MS Bioworks, LLC (www.msbioworks.com) with a Waters NanoAcquity HPLC system interfaced to a ThermoFisher LTQ Orbitrap Velos. Peptides were loaded on a trapping column and eluted over a 75 µm analytical column at 350 nL/min; both columns were packed with Jupiter Proteo resin (Phenomenex). The mass spectrometer was operated in data-dependent mode, with MS performed in the Orbitrap at 60,000 FWHM resolution and MS/MS performed in the LTQ. Peptides were identified that represented 98% of the predicted sequence. A second analysis by higher energy collision induced dissociation produced 48 unique peptides and 545 total spectra. There were 56 phosphopeptide spectra of which 14 were unique. All runs were analyzed by Scaffold (Proteome Software) for validation, filtering, and to create a non-redundant list per sample.

### Purification of polysaccharide capsule and dot-blot analysis

Bacteria were grown overnight without shaking at 30°C in NaCl-free LB media supplemented with 20 mM (NH_4_)_2_SO_4_ and 5 mM MgSO_4_. Cultures were diluted 1∶2500 into 100 ml LB in a 500 ml flask and incubated overnight under the same conditions. Cells were harvested by centrifugation at 4,000 rpm (3,220 g) for 15 min at room temperature. Pellets were washed with 20 ml of phosphate-buffered saline (PBS), resuspended in 1 ml PBS, transferred to 2 ml Eppendorf tubes, centrifuged at 5,000 rpm (2,655 g) for 5 min at room temperature, and finally suspended in 0.9 ml of PBS. Then, an equal volume of phenol pH 8 was added and vigorously shook to mix the phases. Samples were incubated at 70°C for 1 hr (mixing every 5 min) and then centrifuged at 14,000 rpm (12,000 g) at room temperature for 1 hr. The aqueous fraction was phenol extracted three more times. Then samples were extracted twice with an equal volume of chloroform and centrifuged for 30 min at 14,000 rpm (12,000 g). The upper, aqueous phase was recovered, two volumes of 100% ethanol were added to each sample, and the polysaccharides was allowed to precipitate for 1 h at −70°C. Next, the samples were centrifuged at 12,000 g for 30 min, after which the pellets were washed with 500 µl of 70% ethanol, recentrifuged, and dried. Pellets were dissolved in 0.6 ml water at 65°C. The dissolved capsular and LPS material were transferred to ultracentrifuge tubes (Beckman 357448) and centrifuged at 55,000 rpm (186,000 g) in a Beckman TL-100 microcentrifuge (TLA-55 rotor) at 4°C for 2 h with controlled speed decline to pellet the LPS micelles. The top 400 µl supernatant, containing capsular polysaccharide, was carefully transferred to a new tube and the volume was reduced to 50 µl by centrifugal vacuum concentration (SpeedVac). Of the remaining 200 µl, the top ∼140 µl was discarded and the bottom ∼40 µl containing LPS was kept. Samples were run on 10% SDS-PAGE gels followed by Western blot with anti-O127 antiserum to analyze the samples for LPS (2.5 µl per lane). The capsule polysaccharide samples were serially diluted and 2.5 µl from each dilution were dotted on PVDF membranes for Western blots. To ensure that the capsular polysaccharide samples were not contaminated with LPS, 10 µl of the undiluted material was also analyzed by SDS-PAGE and Western blot. Immunoblots were blocked (2% BSA and 2% skimmed milk), and developed with rabbit OK anti-O127 antiserum (Statens Serum Institute) followed by a secondary anti-rabbit IgG antibody conjugated to alkaline phosphatase (Sigma A3687) and stained using the BCIP/NBT reagents (Promega S3771).

### Purification of MBP-Etk(446–726)

Ten milliliters of an overnight culture of BL21(DE3) previously transformed with pKS2084 were diluted into 1 liter of LB-Amp, grown to OD_600_≈0.6 at 37°C, and cooled to 22°C with shaking. IPTG (0.2 mM) was added and shaking continued at 22°C for 16–18 hrs. After centrifugation, cells from 0.5 liter of culture were resuspended in 30 ml buffer A (300 mM NaCl, 20 mM Tris-HCl pH 8) containing one-half tablet of EDTA-free Protease Inhibitor Cocktail (Roche). After lysis by sonication and centrifugation at 48,000 g, the supernatant was filtered and loaded on an 8 ml Talon Superflow (Clontech) column and eluted with a gradient of 300 mM imidazole in buffer A. Fractions containing the fusion protein were pooled, dialyzed against buffer A containing 10% glycerol, concentrated, and frozen at −80°.

### Determination of Etp activity using Etk as substrate

6His-Etp, 6His-EtpY121F, or 6His-EtpD119E were expressed from BL21(DE3) containing pMS0125, pAJ0049, or pAJ2090 by overnight incubation at 22°C. After chromatography of the soluble fraction with Talon Superflow, Etp-containing fractions were dialyzed against phosphate-buffered saline containing 2 mM TCEP and 10% glycerol, and frozen at −80°C. Purified MBP-Etk(446–726) was completely phosphorylated by incubation with ATP and MgCl_2_ and to serve as substrate. Immediately before assay, Etp and Etk samples were buffer exchanged into 200 mM NaCl, 2 mM TCEP, 20 mM Tris-HCl, pH 7.5 by gel filtration in order to remove all phosphate. Dephosphorylation assays in 96-well trays monitored the release of inorganic phosphate by the EnzChek enzyme-linked assay (Invitrogen). Reactions were set up in 96-well trays at 37°C by mixing 50 µl 2-amino-6-mercapto-7-methylpurine riboside, 2.5 µl purine nucleoside phosphorylase, 187.5 µl MBP-Etk substrate at varying concentrations (0–50 µM) in Tris-HCl buffer (pH 7.5), and 10 µl Etp (5–25 µM). Data were fit to the Michaelis-Menton equation with DeltaGraph (Red Rock Software) to derive the kinetic constants.

## Supporting Information

Figure S1 **Etp autodephosphorylation.** To prepare Etp, *E. coli* XL1Blue containing pAP406 (p6His-Etp) was grown in LB at 37°C to OD_600_ = 0.5. After transfer of culture to 20°C for 30 min, IPTG was added to a final concentration of 0.1 mM and the culture was incubated 15 h at 20°C without shaking. The 6His-tagged Etp was extracted and purified under native conditions using Talon metal affinity chromatography according to the protocols recommended by the manufacturer (Clontech). (A) Purified Etp was incubated at pH 6 at 37°C. Aliquots were removed at 0, 1, 5, and 10 min, mixed immediately with SDS loading buffer, and boiled. The phosphorylation levels were tested by Western blot analysis with anti-PY antibody. In (B), equal amounts of purified and partially phosphorylated Etp were loaded into four lanes and blotted onto nitrocellulose membrane. The membrane was then sliced and different strips were incubated in blocking solutions adjusted to different pH levels (6, 7, 8, and 9) for 6 hours. The membranes were then washed and developed with anti-PY antibody. At pH 6 the signal was not detected indicating that Etp was auto-dephosphorylated. A similar experiment is shown in (C) but in this case all the blots were incubated at pH 6 and the incubation time with the blocking solution was varied (1, 2 and 3 h).
(TIF)

Figure S2 **Etp activity at different pHs.** Recombinant 6His-Etp was purified as described in Fig. S1. In (A) the purity was examined by running a SDS-PAGE gel followed by staining with Coomassie Blue. Only one band was visualized (A, left lane). Molecular weight markers are shown in the right lane. In (B) the activity of the enzyme was determined under different pH conditions, using *p*-nitrophenol phosphate (pNPP) as substrate. Maximal activity was observed at pH 6, while the activity at pH 8 was very slow. Phosphatase activity was monitored at 37°C, using a continuous method based on the detection of *p*-nitrophenol formed from pNPP. Dephosphorylation rates were determined at 405 nm in a reaction buffer containing 0.4% PNPP and 100 mM Tris-HCl at the desired pH. The assay was optimized with respect to protein concentration, time, and pH.
(TIF)

Figure S3 **Phenylphosphate inhibits binding of anti-PY antibody to Etp.** 6His-Etp was expressed in EPEC and purified under alkali conditions as described in Fig. S1. The purified protein was used for immunoblotting and reacted with anti-6His (left panel), anti-PY (central panel) and anti-PY in the presence of 10 mM phenylphosphate (the right panel), a competitive inhibitor of the interaction of anti-PY with phosphorylated tyrosine residues. As shown, phenylphosphate completely inhibited binding of the anti-PY antibody (right panel).
(TIF)
