# Supplementary figures and images for: Cycling of Etk and Etp Phosphorylation States Is Involved in Formation of Group 4 Capsule by Escherichia coli
Source: PLoS One. 2012 Jun 4;7(6):e37984. doi: 10.1371/journal.pone.0037984 (PMC3366997; doi:10.1371/journal.pone.0037984)

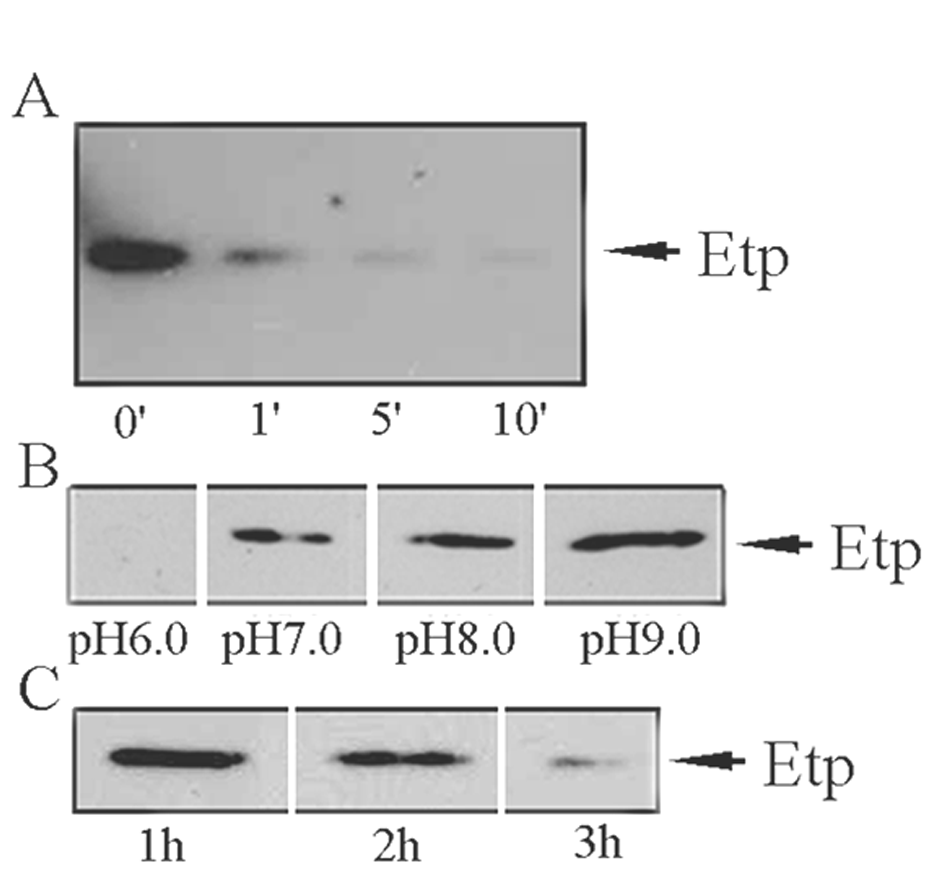

Supplement: Figure S1 — Etp autodephosphorylation. To prepare Etp, E. coli XL1Blue containing pAP406 (p6His-Etp) was grown in LB at 37°C to OD600 = 0.5. After transfer of culture to 20°C for 30 min, IPTG was added to a final concentration of 0.1 mM and the culture was incubated 15 h at 20°C without shaking. The 6His-tagged Etp was extracted and purified under native conditions using Talon metal affinity chromatography according to the protocols recommended by the manufacturer (Clontech). (A) Purified Etp was incubated at pH 6 at 37°C. Aliquots were removed at 0, 1, 5, and 10 min, mixed immediately with SDS loading buffer, and boiled. The phosphorylation levels were tested by Western blot analysis with anti-PY antibody. In (B), equal amounts of purified and partially phosphorylated Etp were loaded into four lanes and blotted onto nitrocellulose membrane. The membrane was then sliced and different strips were incubated in blocking solutions adjusted to different pH levels (6, 7, 8, and 9) for 6 hours. The membranes were then washed and developed with anti-PY antibody. At pH 6 the signal was not detected indicating that Etp was auto-dephosphorylated. A similar experiment is shown in (C) but in this case all the blots were incubated at pH 6 and the incubation time with the blocking solution was varied (1, 2 and 3 h). (TIF) [file pone.0037984.s001.tif]

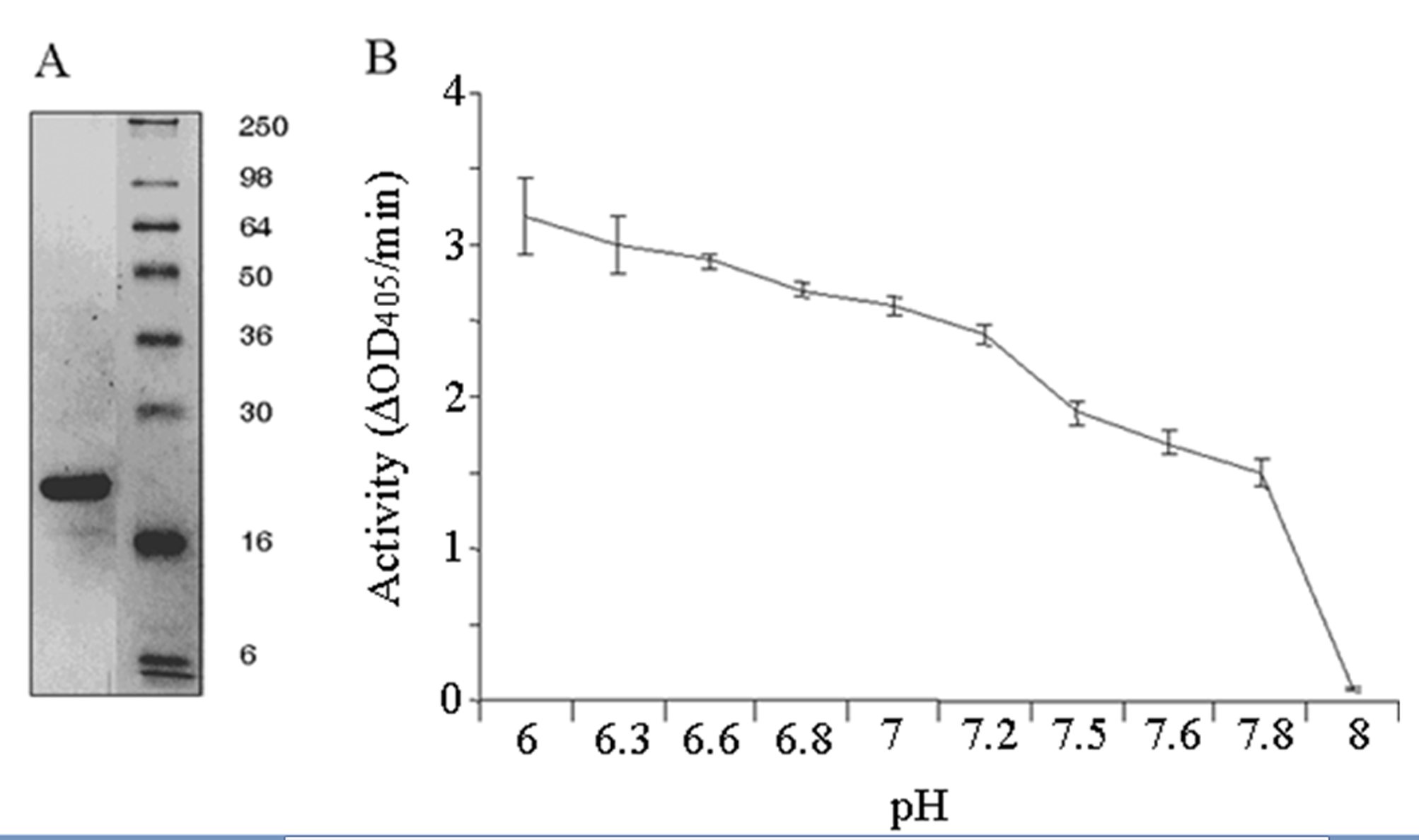

Supplement: Figure S2 — Etp activity at different pHs. Recombinant 6His-Etp was purified as described in Fig. S1. In (A) the purity was examined by running a SDS-PAGE gel followed by staining with Coomassie Blue. Only one band was visualized (A, left lane). Molecular weight markers are shown in the right lane. In (B) the activity of the enzyme was determined under different pH conditions, using p-nitrophenol phosphate (pNPP) as substrate. Maximal activity was observed at pH 6, while the activity at pH 8 was very slow. Phosphatase activity was monitored at 37°C, using a continuous method based on the detection of p-nitrophenol formed from pNPP. Dephosphorylation rates were determined at 405 nm in a reaction buffer containing 0.4% PNPP and 100 mM Tris-HCl at the desired pH. The assay was optimized with respect to protein concentration, time, and pH. (TIF) [file pone.0037984.s002.tif]

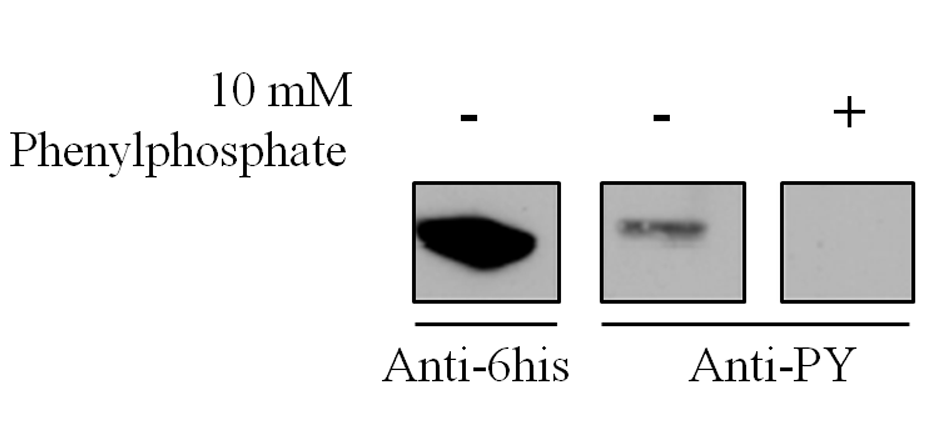

Supplement: Figure S3 — Phenylphosphate inhibits binding of anti-PY antibody to Etp. 6His-Etp was expressed in EPEC and purified under alkali conditions as described in Fig. S1. The purified protein was used for immunoblotting and reacted with anti-6His (left panel), anti-PY (central panel) and anti-PY in the presence of 10 mM phenylphosphate (the right panel), a competitive inhibitor of the interaction of anti-PY with phosphorylated tyrosine residues. As shown, phenylphosphate completely inhibited binding of the anti-PY antibody (right panel). (TIF) [file pone.0037984.s003.tif]
